# Systematic Review on the Impact of Mobile Applications with Augmented Reality to Improve Health

**DOI:** 10.3390/bioengineering11060622

**Published:** 2024-06-18

**Authors:** Beatriz Piqueras-Sola, Jonathan Cortés-Martín, Raquel Rodríguez-Blanque, María José Menor-Rodríguez, Elena Mellado-García, Carolina Merino Lobato, Juan Carlos Sánchez-García

**Affiliations:** 1 Virgen de las Nieves University Hospital, 18014 Granada, Spain; bpiquerassola@gmail.com; 2 Department of Nursing, Faculty of Health Sciences, University of Granada, 18071 Granada, Spain; jcortesmartin@ugr.es (J.C.-M.); e.elenamellado@go.ugr.es (E.M.-G.); jsangar@ugr.es (J.C.S.-G.); 3 San Cecilio University Hospital, 18071 Granada, Spain; carolina.merino.sspa@juntadeandalucia.es; 4 Área Sanitaria Santiago de Compostela-Barbanza, 15706 Santiago de Compostela, Spain; mariajosemenor@hotmail.com

**Keywords:** mobile applications, augmented reality, physical fitness

## Abstract

Physical inactivity represents a significant public health challenge globally. Mobile applications, particularly those utilizing augmented reality (AR), have emerged as innovative tools for promoting physical activity. However, a systematic evaluation of their efficacy is essential. This systematic review aims to evaluate and synthesize the evidence regarding the effectiveness and benefits of mobile applications with augmented reality in enhancing physical activity and improving health outcomes. A comprehensive search was conducted in Scopus, PubMed, WOS, and the Cochrane Library databases following PRISMA guidelines. Observational and interventional studies evaluating AR mobile applications for physical exercise were included, without restrictions on publication date or language. The search terms included “Mobile Applications”, “Augmented Reality”, “Physical Fitness”, “Exercise Therapy”, and “Health Behavior”. The methodological quality was assessed using the ROBINS tool. The review identified twelve eligible studies encompassing 5,534,661 participants. The findings indicated significant increases in physical activity and improvements in mental health associated with the use of AR applications, such as Pokémon GO. However, potential risk behaviors were also noted. The evidence suggests that AR interventions can effectively promote physical activity and enhance health. Nonetheless, further research is needed to address limitations and optimize their efficacy. Future interventions should be tailored to diverse cultural contexts to maximize benefits and mitigate risks. AR mobile applications hold promise for promoting physical activity and improving health outcomes. Strategies to optimize their effectiveness and address identified risks should be explored to fully realize their potential.

## 1. Introduction

In the past decade, technology has revolutionized various aspects of daily life, including health promotion. Among the most notable innovations are mobile applications that incorporate augmented reality (AR), which offer new possibilities for fostering physical activity in an accessible and engaging manner. AR overlays digital information onto the physical environment, creating interactive experiences that can motivate and guide users in their exercise routines.

The intrinsic connection between physical activity and health is irrefutable, with an extensive range of benefits encompassing cardiovascular, physical, and mental well-being. Physical activity plays a crucial role in promoting cardiovascular health by strengthening the heart and improving blood circulation, thereby reducing the risk of heart diseases. Moreover, beyond its positive effects on physical health, physical activity is also closely related to mental health, as regular participation in these activities helps reduce symptoms of depression and anxiety, fostering a state of emotional well-being. Notably, the relationship between physical activity and mortality is evident, with insufficiently active individuals facing a significantly higher risk of death compared to those who maintain an adequate level of physical activity. In this context, these findings highlight the importance of promoting physical activity as a comprehensive measure to improve health and prevent various physical and mental illnesses [1].

For adults aged 18 to 64 years, the World Health Organization (WHO) sets specific guidelines that emphasize the importance of physical activity. It recommends engaging in moderate aerobic physical activities for at least 150 to 300 min per week, or vigorous aerobic physical activities for at least 75 to 150 min per week. This group includes women during pregnancy and the postpartum period, for whom it is advised to engage in moderate aerobic physical activities for at least 150 min per week, incorporating a variety of aerobic and muscle-strengthening activities [2].

In the contemporary era, technology has permeated various aspects of our lives, transforming how we approach different activities, from communication to health. In the realm of promoting a healthy lifestyle, mobile applications have gained prominence as accessible and versatile tools. This research delves into the universe of mobile applications aimed at physical exercise, specifically exploring those that incorporate augmented reality for visualizing exercise videos [3,4].

The growing prevalence of sedentary lifestyles and concerns related to physical and mental health have driven the search for innovative solutions. In this context, mobile applications emerge as potential allies in promoting physical activity and general well-being [2]. These tools, readily available to users, offer a unique opportunity to encourage participation in physical activities from the comfort of home [2,5,6,7].

The use of mobile AR applications in the realm of physical exercise has shown significant potential for increasing physical activity and improving both the mental and physical health of users [8,9,10]. Recent studies have demonstrated that applications like Pokémon GO not only increase physical activity but also contribute to improvements in mental well-being, reducing symptoms of depression and anxiety [1]. Additionally, these applications allow the integration of playful and social elements, which can enhance motivation and long-term commitment to physical activity.

The objective of this systematic review is to evaluate and synthesize the evidence on the effectiveness and benefits of mobile applications with augmented reality in promoting physical exercise and improving health. This analysis focuses on three fundamental dimensions: the enhancement of physical activity levels, the overall impact on users’ health, and the capacity of these applications to foster adherence to proposed exercise programs. Through this review, we aim not only to explore the potential of AR in the realm of exercise but also to identify strategies to optimize its effectiveness and address any identified risks.

## 2. Materials and Methods

The methodology employed for the preparation of this report involved conducting a systematic review of the available scientific literature on the use of mobile applications that utilize augmented reality for the visualization of exercise videos among smartphone users. This process closely adhered to the Preferred Reporting Items for Systematic reviews and Meta-Analyses (PRISMA) review protocol which was followed nearly comprehensively.

The review protocol has been registered on the website: http://www.crd.york.ac.uk/PROSPERO/, with the registration identification number CRD42024509239 (accessed on 12 February 2024).

A research question was formulated using the PICO framework with the following terms:

- Population: Users of mobile applications designed to improve health through physical exercise.
- Intervention: Mobile applications incorporating augmented reality for the visualization of exercise videos.
- Comparison: Not applicable in this context, as there is no comparison with another intervention.
- Outcome: Evaluation of effectiveness and benefits in terms of physical activity, to improve health.

The formulated research question is:

“What is the impact of augmented reality mobile applications on improving levels of physical exercise, the overall health of users, and adherence to exercise programs?”

During the article selection process, specific requirements were established regarding methodology, excluding systematic reviews, editorials, letters to the editor, books, and comments. There were no restrictions on publication date or language. The selection favored articles aligned with the research theme and encompassing research articles, including descriptive studies or clinical trials.

The literature search was conducted in the Scopus, PubMed, WOS (Web of Science), and Cochrane Library databases. The structured language used was derived from MeSH terms and Health Sciences Descriptors (DeCS). The descriptors used are reflected in Appendix A. Boolean operators AND and OR were employed in the search process.

In Appendix B, the search strategy used for this study is presented. Searches in SCOPUS, PubMed, WOS, and Cochrane Library were conducted in June 2024. After implementing the search strategy, the located articles were transferred to the Mendeley web application using the Mendeley Web Importer tool. Subsequently, they were organized into folders according to the original database, and duplicate articles were removed.

The title, abstract, and keywords of each study identified in the search were reviewed, ensuring that the article’s subject matter aligned with the research objectives. Those studies considered potentially eligible were thoroughly examined.

During this phase, data related to study quality, user characteristics, interventions, and relevant outcomes were analyzed. This thorough review process ensured the inclusion of studies that were both relevant and consistent with the goals of the study.

The following data were extracted from each included article: participant characteristics (number of participants and age) and intervention characteristics (location and type of intervention), and finally, the conclusions were drawn. A more detailed explanation of the article selection process is provided in the Results Section.

To assess the risk of bias in the selected observational studies, the ROBINS tool (Risk Of Bias In Non-randomized Studies of Interventions) was employed. This tool is utilized to evaluate the methodological quality and risk of bias in non-randomized studies of interventions, including observational and quasi-experimental studies.

Based on the information provided by this review, a series of premises are derived as results, which will serve to standardize concepts regarding the use of augmented reality on mobile devices for physical exercise.

## 3. Results

The execution of this systematic review has been a meticulous process aimed at synthesizing and comprehensively evaluating the available scientific evidence to address the research question. Within this framework, the article selection has played a crucial role, identifying and subsequently including relevant studies essential for the integrity and validity of the review.

**Figure 1:**
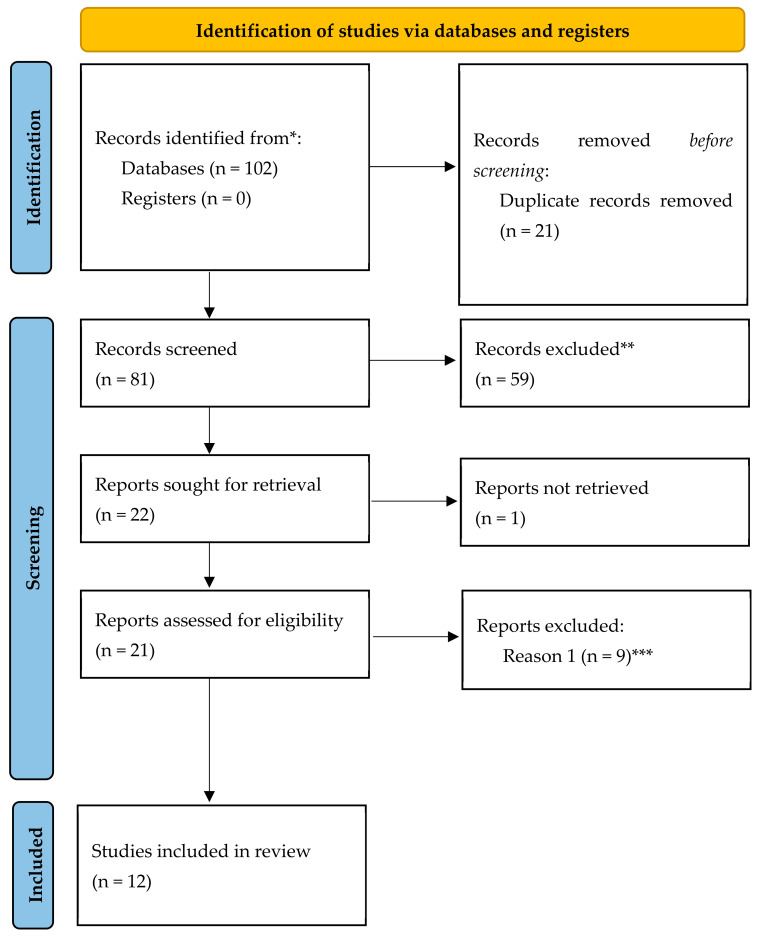
Flowchart of article selection. * A total of 102 studies were identified, with 59 housed in the SCOPUS database, 24 in WOS, 18 in PubMed, and 1 in Cochrane Library. ** Studies that utilized augmented reality for student learning, professional applications, disease monitoring, prototypes of augmented reality devices, and articles with methodologies outside the inclusion criteria were excluded. *** Ultimately, does not use augmented reality for physical exercise.

To provide a clear and transparent overview of this process, a flowchart (Figure 1) has been developed, illustrating each stage of the article selection process.

All selected articles underwent a risk of bias assessment using the ROBINS tool. The results are presented in detail and organized in Table 1, breaking down the data according to the different domains assessed. Each domain offers a specific insight into potential biases in the study, thus allowing for a more comprehensive understanding of the methodological quality and reliability of the obtained results.

In summary, the studies exhibit a low risk of bias in most evaluated domains, except for the confounding variables domain where the risk is high due to inadequate control of confounding factors. Overall, the methodological quality is deemed adequate.

A detailed assessment of the risk of bias in the studies included in this systematic review provides a comprehensive overview indicating that, in general, these studies demonstrate methodological features comparable to those found in randomized clinical trials. The application of the criteria reflects a significant effort by researchers to ensure the methodological robustness and internal validity of their respective studies.

A cohort of 12 articles meeting predefined selection criteria and aligning with the research question was identified. Next, a detailed analysis of each selected article will be presented, outlining their individual characteristics and contributions to the research in question (Table 2).

## 4. Discussion

The cohort of 12 studies provides a comprehensive evaluation of various augmented reality (AR) interventions aimed at promoting physical activity and health across diverse populations. The interventions range from popular mobile games like Pokémon GO to specialized applications designed for specific demographics, such as children or athletes.

### 4.1. Positive Impacts on Physical and Social Health

Yip et al. [11] and Xian et al. [20] both explore the effects of playing Pokémon GO. Yip et al. observed increased physical activity and social interactions among young adults in Hong Kong, though they noted risks like game addiction and accidents. Similarly, Xian et al.’s multinational study reported significant increases in physical activity, averaging 2000 additional steps per day. Both studies underscore the dual benefits of physical and social health improvements but also highlight associated risks, such as playing while in moving vehicles, which must be mitigated.

### 4.2. Mental Health Benefits

Watanabe et al. [12] focused on the psychological benefits of playing Pokémon GO among a large sample of workers in Japan. The study found a significant reduction in psychological distress, suggesting a potential population-level impact on mental health. However, no notable differences were found in physical complaints or work performance, indicating that while mental health benefits are evident, physical health impacts in a work setting require further exploration.

### 4.3. Motivation and Long-Term Engagement

The motivational aspects of AR games were explored by Rasche et al. [13], who identified factors that influence the initiation and continuation of playing Pokémon GO. Social interactions emerged as a critical component for long-term engagement, highlighting the game’s ability to foster sustained physical activity through social connectivity.

### 4.4. Targeted Interventions

Intawong et al. [14] examined the effectiveness of a gamified AR app, “Camt comic run”, at Chiang Mai University. The study revealed increased participation and motivation among participants with initially low physical activity levels but did not significantly affect those already meeting activity guidelines. This indicates that gamification can be a powerful tool for specific groups but may have limited impact on those already active.

### 4.5. Adolescent Engagement and Virtual Reality

Farič et al. [15] and Farič et al. [16] investigated the potential of virtual reality (VR) and narrative-based AR applications. The former found that VR shows promise in engaging adolescents in physical activity, although further development is needed. The latter study highlighted the effectiveness of narrative-based AR applications like “Zombies Run” in altering perceptions of exercise and promoting healthy behaviors. These findings suggest that immersive and narrative-driven approaches can significantly enhance engagement and motivation among younger populations.

### 4.6. Social and Environmental Awareness

Escaravajal-Rodríguez et al. [17] studied Pokémon GO users in Spain, noting the game’s positive role in promoting physical activity, social relationships, and environmental awareness. These results indicate that AR games can extend benefits beyond physical health, contributing to broader social and ecological engagement.

### 4.7. Cultural and Contextual Adaptations

Alturki et al. [18] emphasized the importance of culturally tailored interventions with their study on the “Akser Waznk” app in Saudi Arabia. The findings suggest that mobile technology can effectively change unhealthy behaviors, but success depends on considering the target group’s social and cultural norms.

### 4.8. Safety and Accessibility

Barbero et al. [19] evaluated the safety of playing Pokémon GO among a diverse population in Northern California. They found that the risks were comparable to other mild to moderate physical activities, with a low incidence of serious injuries. This suggests that AR games can be a safe and accessible way to promote physical activity among sedentary populations.

### 4.9. Specialized Interventions for Children and Athletes

Ahn et al. (2024) [21] and Usra et al. (2024) [22] focused on specific demographic groups. Ahn et al. implemented the “Virtual Fit Buddy” ecosystem among children, finding it effective in reducing sedentary behavior and increasing light-intensity physical activity, especially in less active children and those with pets. Usra et al. evaluated an AR training program for female athletes in combat sports, concluding that the AR program significantly improved both physical fitness and technical performance compared to traditional training methods.

The reviewed studies exhibit several limitations impacting their generalizability and robustness. Small sample sizes and geographic constraints limit the applicability of findings to broader populations. Short-term study designs and a lack of control groups, as seen in some studies, hinder the ability to draw definitive conclusions about long-term effects. Reliance on self-reported data introduces potential biases, while a focus on specific demographics restricts generalizability. Measurement limitations and the need for further development of AR technologies highlight the preliminary nature of these interventions. These issues underscore the necessity for future research with larger, more diverse samples, rigorous control mechanisms, and comprehensive health assessments to strengthen the evidence base for AR interventions in public health [11,12,13,17,18,20].

## 5. Conclusions

This systematic review of 12 studies on augmented reality (AR) interventions reveals promising evidence in promoting physical activity and health across diverse populations. Significant benefits are observed in physical, social, and mental health, although limitations such as small sample sizes, short-term study design, and potential risks like gaming addiction are identified. Further research with robust designs and representative samples is needed to fully comprehend the long-term impact and ensure the effectiveness and safety of these interventions in public health, emphasizing the importance of cultural and contextual adaptation for successful implementation. The conclusion requires a comprehensive overhaul to better underscore the imperative to explicitly address associated risks and cultural adaptations. Moreover, exploring strategies to optimize the effectiveness of these technologies could significantly enhance the article’s impact. Future research should focus on addressing the identified limitations and consider suggestions such as longer intervention periods, more rigorous control mechanisms, and comprehensive health assessments to strengthen the evidence base for AR interventions in public health.

## Figures and Tables

**Table 1:** Risk of bias assessment according to the ROBINS Scale.

Author	Participant Selection Domain	Exposure Classification Domain	Unmediated Confusion Domain	Predicted Exposure Deviation Domain	Result Classification Domain	Missing Data Domain	General Domain
Yip et al. (2023) [11]	Low risk. Purposive sampling was used but with predefined criteria	Low risk. The intervention was to participate in the Pokémon GO game	Low risk. There were no significant confounders.	Low risk. Exposure was measured directly by game participation	Low risk. The results were based on qualitative interviews	Low risk. There were no missing data	Low risk. It is a qualitative study with a low risk of bias in general.
Watanabe et al. (2017) [12]	Low risk, it was non- random sampling but with predefined criteria.	Low risk, exposure was measured directly	Low risk, no important confounding factors reported	Low risk, exposure was measured directly	Low risk, results were based on validated self-report scales	No risk, no missing data	Low risk, it is an observational study with low risk of bias in general
Rasche et al. [13]	Uncertain risk, it was a non-probabilistic open web survey	Low risk, exposure was measured directly by game use	Low risk, no important confounding factors reported	Low risk, exposure was measured directly	Low risk, results were based on validated surveys	No risk, no lost data reported	Uncertain risk given the non-probabilistic open survey design and web recruitment
Intawong et al. (2021) [14]	Low risk, it was non- random sampling but with predefined criteria.	Low risk of bias. There is no evidence that the inclusion/exclusion criteria led to systematic bias	Low risk of bias. The intervention was clearly defined	Low risk of bias. There are no differences in the measurement of results between the groups	Low risk of bias. There are no significant differences in dropout rates between the groups.	Low risk of bias. The groups were appropriately compared on prognostic factors.	Low risk of bias. The measurement of the results was carried out in the same way for all participants.
Faric et al. (2021) [15]	Low risk of bias. It appears that there were no selection factors that led to bias	High risk of bias. There was no random assignment of participants	Low risk of bias. The intervention is well defined	Low risk of bias. There seems to be no change in the intervention	Low risk of bias. There do not seem to be differences in the measurement of results	Low risk of bias. There do not appear to be significant differences in dropout rates.	Uncertain risk of bias. Lack of information for some domains
Farič et al. (2021) [16]	Low risk of bias. Participants were randomly selected from users willing to be interviewed.	Low risk of bias. All participants were exposed to the same “intervention” which was the use of the Zombies app, Run!	Low risk of bias. There do not appear to be unmeasured confounders.	Low risk of bias. The “exposure” was consistent for all participants	Low risk of bias. The results were measured in the same way for everyone through qualitative interviews	Low risk of bias. There do not appear to be significant missing data	Low risk of bias
Escaravajal -Rodríguez (2018) [17]	Low risk of bias. Participants were randomly selected from users willing to be interviewed.	Low risk of bias. All participants were exposed to the same “intervention” which was the use of the Pokémon GO application	Low risk of bias. There do not appear to be unmeasured confounders.	Low risk of bias. There seems to be no change in the “intervention”	Low risk of bias. The results were measured through qualitative interviews in a consistent manner	Low risk of bias. There do not appear to be significant differences in dropout rates.	Uncertain risk of bias. Lack of information to evaluate some domains
Alturki et al. (2019) [18]	Low risk of bias. The participants were selected randomly	Low risk of bias. They were all exposed to the same “intervention”	Low risk of bias. There do not appear to be unmeasured confounders.	Low risk of bias. There seems to be no change in the “intervention”	Low risk of bias. Results were measured consistently	Low risk of bias. There do not appear to be significant missing data	Low risk of bias
Barbero et al. (2018) [19]	Low risk. All Kaiser Permanente Northern California patients who came for consultation for Pokémon GO were included.	Low risk. The exposition was clearly defined (playing Pokémon Go)	Moderate risk. It was not adjusted for socioeconomic determinants that could influence the results	Low risk. Exposure was constant throughout follow-up	Low risk. The results are well defined and classified	Tracking appears complete	Low risk of bias
Xian et al. (2017) [20]	Low risk. Participants voluntarily self-enrolled in the study and met pre-established selection criteria. There does not appear to be bias in the allocation of participants.	Low risk. Exposure (playing Pokémon GO) was measured directly without ambiguity. All participants were exposed to the same intervention	High risk. It was not statistically adjusted for confounding variables such as socioeconomic level, marital status, place of residence or others that could influence the results.	Low risk. Exposure was consistent for all participants as it was measured before and after the start of the game.	Low risk. The results (daily steps counted by the iPhone app) were measured automatically and objectively without ambiguity.	Low risk. There do not appear to be any missing data or participants excluded from the analyses. The follow-up was complete.	Low risk. The tool used to measure outcomes (iPhone health app) has not been widely validated, but each participant served as their own control
Ahn, et al. (2024) [21]	Low risk. The study included 303 parent/child pairs recruited from 19 schools and YMCA branches in the metropolitan area of Atlanta, Georgia. Random assignment was balanced between groups.	Low risk. Consistent classification of exposure (participation in the VFB ecosystem) using Fitbit and Actigraph devices to monitor physical activity.	Low risk. Models were adjusted for several factors (accelerometer usage time, child overweight status, sex, race, age, and primary caregiver’s education level).	Low risk. Modifications between cohorts to improve fidelity and acceptance, with consistent effects observed in both groups.	Low risk. Outcomes measured with validated methods (Fitbit, Actigraph) and analyzed with hierarchical mixed linear models to handle data correlation.	Low risk. No significant issues with missing data reported, and appropriate methodologies applied to handle any missing data.	Low risk. Rigorous implementation of methodologies, balanced random assignment, and adequate adjustment for confounding factors indicate a low overall risk of bias.
Usra et al. (2024) [22]	Low risk. Participants were female athletes from Pencak Silat and Karate, selected based on specific inclusion and exclusion criteria. Randomization was used to allocate them into experimental and control groups.	Low risk. Clear definitions and classifications of exposure were provided, distinguishing between AR and non-AR training programs.	Moderate risk. While some potential confounders were addressed, not all possible confounding variables were explicitly controlled.	Low risk. There was a clear adherence to the planned interventions with no significant deviations reported.	Low risk. Technical performance and physical fitness were measured using standardized and validated instruments.	Low risk. The study accounted for missing data appropriately and maintained sufficient sample size for statistical power.	Low risk. Overall, the study maintains a low risk of bias across domains.

**Table 2:** Main results obtained.

Study	Sample	Location	Intervention	Findings
Yip et al. [11]	60 participants, aged 18–25 (average 20.9)	Hong Kong, China (2017)	Playing “Pokémon GO”	Positive impacts on physical and social health. Increased physical activity and social interactions. Risks: game addiction and distractions leading to accidents.
Xian et al. [20]	167 participants, aged 18–29 (average 25)	Multinational	Playing “Pokémon GO”	Significant increase in physical activity (2000 additional steps/day). Risks: playing while in moving vehicles. Benefits need risk behaviors to be addressed.
Watanabe et al. [12]	2530 participants, aged 18–65 (average 42)	Japan	Playing “Pokémon GO”	Improvement in psychological distress. No significant differences in physical complaints or work performance. Highlights mental health benefits.
Rasche et al. [13]	199 participants (age unspecified)	Germany	Playing “Pokémon GO”	Identified motivational aspects influencing game engagement. Emphasizes social interactions for long-term motivation.
Intawong et al. [14]	40 participants, aged 18–40	Chiang Mai University, Thailand	“Camt comic run” AR app	Increased participation and motivation among those with low physical activity levels. No significant improvement for those already meeting activity guidelines.
Farič et al. [15]	511 adolescents for quantitative survey; 6423 opened, 5343 completed “Zombies Run” survey	United Kingdom	Virtual reality game prototype	Promising engagement of adolescents in physical activity. Further development and testing needed.
Farič et al. [16]	30 participants, aged 16–71	Multinational	Prolonged use of “Zombies Run”	Effective in engaging physical activity through narrative. Potential to promote healthy behaviors through engaging narratives.
Escaravajal-Rodríguez et al. [17]	714 participants, aged 16–56 (average 24.86)	Spain	Surveys of “Pokémon GO” users	Positive role in promoting physical activity, social relationships, and environmental awareness.
Alturki et al. [18]	26 participants, aged 16–71 (average 33.1)	Saudi Arabia	“Akser Waznk” app trial	Potential to change unhealthy behaviors in Saudi community. Emphasizes need for culturally tailored interventions.
Barbero et al. [19]	394 participants, median age 27 (range 17–42)	Kaiser Permanente Northern California	Playing “Pokémon GO”	Comparable risks to other mild to moderate physical activities. Potential to reach sedentary populations.
Ahn et al. (2024) [21]	303 parent/child pairs (children aged 6–11, mean age 8.1)	Atlanta, Georgia	“Virtual Fit Buddy” ecosystem	Reduced sedentary behavior and increased light-intensity physical activity, especially in less active children and those owning a dog.
Usra et al. (2024) [22]	60 female athletes (Pencak Silat and Karate)	Sriwijaya University, Indonesia	AR training program	Significant improvements in physical fitness and technical performance, outperforming traditional training methods.

## Data Availability

Data are available on request from the corresponding author.

## Appendix A

**Table A1:** MeSH Terms and Synonyms Used in Searches.

MeSH Terms	Synonyms
Mobile Applications	“Application Mobile” OR “Applications Mobile” OR “Mobile Application” OR “Mobile Apps” OR “App Mobile” OR “Apps Mobile” OR “Mobile App” OR “Portable Software Apps” OR “App Portable Software” OR “Portable Software App” OR “Software App Portable” OR “Portable Software Applications” OR “Application Portable Software” OR “Portable Software Application” OR “Software Application Portable” OR “Smartphone Apps” OR “App Smartphone” OR “Apps Smartphone” OR “Smartphone App” OR “Portable Electronic Apps” OR “App Portable Electronic” OR “Electronic App Portable” OR “Portable Electronic App” OR “Portable Electronic Applications” OR “Application Portable Electronic” OR “Electronic Application Portable” OR “Portable Electronic Application”
Augmented Reality	“Augmented Realities” OR “Realities Augmented” OR “Reality Augmented” OR “Mixed Reality” OR “Mixed Realities” OR “Realities Mixed” OR “Reality Mixed”
Physical Fitness	“Fitness Physical” OR “Exercise Therapy” OR “Physical Endurance” OR “Exercise”
Exercise Therapy	“Remedial Exercise” OR “Exercise Remedial” OR “Exercises Remedial” OR “Remedial Exercises” OR “Therapy Exercise” OR “Exercise Therapies” OR “Therapies Exercise” OR “Rehabilitation Exercise” OR “Exercise Rehabilitation” OR “Exercises Rehabilitation” OR “Rehabilitation Exercises”
Health Behavior	“Behavior Health” OR “Behaviors Health” OR “Health Behaviors” OR “Health-Related Behavior” OR “Behavior Health-Related” OR “Behaviors Health-Related” OR “Health Related Behavior” OR “Health-Related Behaviors”

## Appendix B

**Table A2:** Search strategy used in each database.

Equation	Database
*((TITLE-ABS-KEY (mobile AND applications) OR TITLE-ABS-KEY (“Application Mobile” OR “Applications Mobile” OR “Mobile Application” OR “Mobile Apps” OR “App Mobile” OR “Apps Mobile” OR “Mobile App” OR “Portable Software Apps” OR “App Portable Software” OR “Portable Software App” OR “Software App Portable” OR “Portable Software Applications” OR “Application Portable Software” OR “Portable Software Application” OR “Software Application Portable” OR “Smartphone Apps” OR “App Smartphone” OR “Apps Smartphone” OR “Smartphone App” OR “Portable Electronic Apps” OR “App Portable Electronic” OR “Electronic App Portable” OR “Portable Electronic App” OR “Portable Electronic Applications” OR “Application Portable Electronic” OR “Electronic Application Portable” OR “Portable Electronic Application”))) AND ((TITLE-ABS-KEY (augmented AND reality) OR TITLE-ABS-KEY (“Augmented Realities” OR “Realities Augmented” OR “Reality Augmented” OR “Mixed Reality” OR “Mixed Realities” OR “Realities Mixed” OR “Reality Mixed”))) AND (((TITLE-ABS-KEY (health AND behavior) OR TITLE-ABS-KEY (“Behavior Health” OR “Behaviors Health” OR “Health Behaviors” OR “Health-Related Behavior” OR “Behavior Health-Related” OR “Behaviors Health-Related” OR “Health Related Behavior” OR “Health-Related Behaviors”))) OR ((TITLE-ABS-KEY (exercise AND therapy) OR TITLE-ABS-KEY (“Remedial Exercise” OR “Exercise Remedial” OR “Exercises Remedial” OR “Remedial Exercises” OR “Therapy Exercise” OR “Exercise Therapies” OR “Therapies Exercise” OR “Rehabilitation Exercise” OR “Exercise Rehabilitation” OR “Exercises Rehabilitation” OR “Rehabilitation Exercises”))) OR ((TITLE-ABS-KEY (physical AND fitness) OR TITLE-ABS-KEY (“Fitness Physical” OR “Exercise Therapy” OR “Physical Endurance” OR “Exercise”)))) AND (LIMIT-TO (DOCTYPE, “ar”))*	Scopus
(((“mobile applications”[MeSH Terms]) OR (“Application Mobile” OR “Applications Mobile” OR “Mobile Application” OR “Mobile Apps” OR “App Mobile” OR “Apps Mobile” OR “Mobile App” OR “Portable Software Apps” OR “App Portable Software” OR “Portable Software App” OR “Software App Portable” OR “Portable Software Applications” OR “Application Portable Software” OR “Portable Software Application” OR “Software Application Portable” OR “Smartphone Apps” OR “App Smartphone” OR “Apps Smartphone” OR “Smartphone App” OR “Portable Electronic Apps” OR “App Portable Electronic” OR “Electronic App Portable” OR “Portable Electronic App” OR “Portable Electronic Applications” OR “Application Portable Electronic” OR “Electronic Application Portable” OR “Portable Electronic Application”)) AND ((“augmented reality”[MeSH Terms]) OR (“Augmented Realities” OR “Realities Augmented” OR “Reality Augmented” OR “Mixed Reality” OR “Mixed Realities” OR “Realities Mixed” OR “Reality Mixed”))) AND ((((“exercise therapy”[MeSH Terms]) OR (“Remedial Exercise” OR “Exercise Remedial” OR “Exercises Remedial” OR “Remedial Exercises” OR “Therapy Exercise” OR “Exercise Therapies” OR “Therapies Exercise” OR “Rehabilitation Exercise” OR “Exercise Rehabilitation” OR “Exercises Rehabilitation” OR “Rehabilitation Exercises”[MeSH Terms])) OR ((“health behavior”[MeSH Terms]) OR (“Behavior Health” OR “Behaviors Health” OR “Health Behaviors” OR “Health-Related Behavior” OR “Behavior Health-Related” OR “Behaviors Health-Related” OR “Health Related Behavior” OR “Health-Related Behaviors”))) OR ((“physical fitness”[MeSH Terms]) OR (“Fitness Physical” OR “Exercise Therapy” OR “Physical Endurance” OR “Exercise”[MeSH Terms])))	Pubmed
(#1 AND #2 AND #7) AND (DT==(“ARTICLE”)) #1 TS=(Mobile Applications) OR TS=(“Application Mobile” OR “Applications Mobile” OR “Mobile Application” OR “Mobile Apps” OR “App Mobile” OR “Apps Mobile” OR “Mobile App” OR “Portable Software Apps” OR “App Portable Software” OR “Portable Software App” OR “Software App Portable” OR “Portable Software Applications” OR “Application Portable Software” OR “Portable Software Application” OR “Software Application Portable” OR “Smartphone Apps” OR “App Smartphone” OR “Apps Smartphone” OR “Smartphone App” OR “Portable Electronic Apps” OR “App Portable Electronic” OR “Electronic App Portable” OR “Portable Electronic App” OR “Portable Electronic Applications” OR “Application Portable Electronic” OR “Electronic Application Portable” OR “Portable Electronic Application”) #2 (TS=(Augmented Reality)) OR TS=(“Augmented Realities” OR “Realities Augmented” OR “Reality Augmented” OR “Mixed Reality” OR “Mixed Realities” OR “Realities Mixed” OR “Reality Mixed”) #7 ((TS=(Health Behavior)) OR TS=(“Behavior Health” OR “Behaviors Health” OR “Health Behaviors” OR “Health-Related Behavior” OR “Behavior Health-Related” OR “Behaviors Health-Related” OR “Health Related Behavior” OR “Health-Related Behaviors”) AND (TS=(Exercise Therapy)) OR TS=(“Remedial Exercise” OR “Exercise Remedial” OR “Exercises Remedial” OR “Remedial Exercises” OR “Therapy Exercise” OR “Exercise Therapies” OR “Therapies Exercise” OR “Rehabilitation Exercise” OR “Exercise Rehabilitation” OR “Exercises Rehabilitation” OR “Rehabilitation Exercises”) AND (TS=(Physical Fitness)) OR TS=(“Fitness Physical” OR “Exercise Therapy” OR “Physical Endurance” OR “Exercise”))	WOS
#1 MeSH descriptor: [Mobile Applications] explode all trees 1607 #2 MeSH descriptor: [Augmented Reality] explode all trees 80 #3 MeSH descriptor: [Physical Fitness] explode all trees 4482 #4 MeSH descriptor: [Exercise Therapy] explode all trees 19822 #5 MeSH descriptor: [Health Behavior] explode all trees 46852 #6 #1AND#2AND(#3OR#4OR#5) 1	COCHRANE LIBRARY

## References

[1] O.P.S./O.M.S Organización Panamericana de la Salud Actividad Física. https://www.paho.org/es/temas/actividad-fisica.

[2] O.M.S Actividad Física. https://www.who.int/es/news-room/fact-sheets/detail/physical-activity.

[3] U.S. Department of Health and Human Services (HHS)—Office of Disease Prevention and Health Promotion Physical Activity Guidelines for Americans Physical Activity Guidelines for Americans. https://health.gov/our-work/nutrition-physical-activity/physical-activity-guidelines.

[4] Escalante Y. (2011). Actividad Física, Ejercicio Físico y Condición Física en El Ámbito de La Salud Pública. Rev. Esp. Salud. Publica.

[5] Casperen C.J., Powell K.E., Christenson G.M. (1985). Physical Activity, Exercise, and Physical Fitness: Definitions and Distinctions for Health-Related Research. Public Health Rep..

[6] Vidal Ledo M., Lío Alonso B., Santiago Garrido A., Muñoz Hernández A., del Morales Suárez I.R., Toledo Fernández A.M. (2017). Realidad Aumentada. Educ. Médica Super..

[7] Ortiz Ortiz J.L., Sepúlveda Gómez A.F. (2019). Aplicación Móvil Que Utiliza Realidad Aumentada Para Apoyar El Aprendizaje Del Acondicionamiento Físico En El Gimnasio Bodytech Sede Diverplaza Ubicado En.

[8] Romeo A., Edney S., Plotnikoff R., Curtis R., Ryan J., Sanders I., Crozier A., Maher C. (2019). Can Smartphone Apps Increase Physical Activity? Systematic Review and Meta-Analysis. J. Med. Internet. Res..

[9] Pradal-Cano L., Lozano-Ruiz C., Pereyra-Rodríguez J.J., Saigí-Rubió F., Bach-Faig A., Esquius L., Xavier Medina F., Aguilar-Martínez A. (2020). Using Mobile Applications to Increase Physical Activity: A Systematic Review. Int. J. Environ. Res. Public Health.

[10] Aznar Díaz I., Cáceres Reche M.P., Trujillo Torres J.M., Romero Rodríguez J.M. (2019). Impacto de Las Apps Móviles En La Actividad Física: Un Meta-Análisis (Impact of Mobile Apps on Physical Activity: A Meta-Analysis). Retos.

[11] Yip Y.C., Yip K.H., Tsui W.K. (2023). Young Adults’ Perspectives on the Implications of an Augmented Reality Mobile Game for Communities’ Public Health: A Qualitative Study. Int. J. Public Health.

[12] Watanabe K., Kawakami N., Imamura K., Inoue A., Shimazu A., Yoshikawa T., Hiro H., Asai Y., Odagiri Y., Yoshikawa E. (2017). Pokémon GO and Psychological Distress, Physical Complaints, and Work Performance among Adult Workers: A Retrospective Cohort Study. Sci. Rep..

[13] Rasche P., Schlomann A., Mertens A. (2017). Who Is Still Playing Pokémon Go? A Web-Based Survey. JMIR Serious Games.

[14] Intawong K., Puritat K. (2021). A Framework of Developing Mobile Gamification to Improve User Engagement of Physical Activity: A Case Study of Location-Based Augmented Reality Mobile Game for Promoting Physical Health. Int. J. Online Biomed. Eng..

[15] Farič N., Smith L., Hon A., Potts H.W.W., Newby K., Steptoe A., Fisher A. (2021). A Virtual Reality Exergame to Engage Adolescents in Physical Activity: Mixed Methods Study Describing the Formative Intervention Development Process. J. Med. Internet. Res..

[16] Farič N., Potts H.W.W., Rowe S., Beaty T., Hon A., Fisher A. (2021). Running App “Zombies, Run!” Users’ Engagement with Physical Activity: A Qualitative Study. Games Health J..

[17] Escaravajal-Rodríguez J.C. (2018). Pokémon GO y Su Influencia En Usuarios Españoles de Facebook. Apunt. Educ. Física Deportes.

[18] Alturki R., Gay V. (2019). The Development of an Arabic Weight-Loss App Akser Waznk: Qualitative Results. JMIR Form. Res..

[19] Barbero E.M., Carpenter D.M., Maier J., Tseng D.S. (2018). Healthcare Encounters for Pokémon Go: Risks and Benefits of Playing. Games Health J..

[20] Xian Y., Xu H., Xu H., Liang L., Hernandez A.F., Wang T.Y., Peterson E.D. (2017). An Initial Evaluation of the Impact of Pokémon GO on Physical Activity. J. Am. Heart Assoc..

[21] LeBlanc A.G., Chaput J.-P. (2017). Pokémon Go: A Game Changer for the Physical Inactivity Crisis?. Prev. Med..

[22] Freeman B., Chau J., Mihrshahi S. (2017). Why the Public Health Sector Couldn’t Create Pokémon Go. Public Health Res. Pract..

